# The development of loop-mediated isothermal amplification (LAMP) assays for the rapid authentication of five forbidden vegetables in strict vegetarian diets

**DOI:** 10.1038/srep44238

**Published:** 2017-03-14

**Authors:** Meng-Shiou Lee, Ting-Ying Su, Yi-Yang Lien, Shyang-Chwen Sheu

**Affiliations:** 1 China Medical University, Department of Chinese Pharmaceutical Science and Chinese Medicine Resources, Taichung, 40402, Taiwan; 2 National Pingtung University of Science and Technology, Department of Food Science, Pingtung, 91201, Taiwan; 3 National Pingtung University of Science and Technology, Department of Veterinary Medicine, Pingtung, 91201, Taiwan

## Abstract

Plant-based food ingredients such as garlic, Chinese leek, Chinese onion, green onion and onion are widely used in many cuisines around the world. However, these ingredients known as the “five forbidden vegetables” (FFVs) are not allowed in some vegetarian diets. In this study, a loop-mediated isothermal amplification (LAMP) assay was developed for the detection of FFVs using five respective LAMP primer sets. The specific primers targeted the ITS1-5.8S-ITS2 nuclear ribosomal DNA sequence regions among the five vegetables. The results demonstrated that the identification of FFVs using the newly developed LAMP assay is more sensitive than the traditional PCR method. Using pepper, basil, parsley, chili and ginger as references, established LAMP primer sets showed high specificity for the identification of the FFV species. Moreover, when FFVs were mixed with other plant ingredients at different ratios (100:0, 50:50, 20:80, 10:90, 5:95, 2:98, and 1:99), no cross-reactivity was evident using LAMP. Finally, genomic DNAs extracted from boiled and steamed FFVs in processed foods were used as templates; the performance of the LAMP reaction was not influenced using validated LAMP primers. Not only can FFV ingredients be identified but commercial foods containing FFVs can also be authenticated. This LAMP method will be useful for the authentication of FFVs in practical food markets in the future.

In recent years, many people have become vegetarian due to rising health concerns^1^. Plant-based foods such as fruits, vegetables and whole grains are believed to be important components of the diet for the prevention of chronic diseases. Experimental evidence has demonstrated that a high consumption of plant-based foods is associated with a significantly lower risk of cardiovascular disease^2^,^3^. The American Dietetic Association (ADA) has reported that vegetarians tend to have several health advantages, including lower cholesterol levels, decreased blood pressure and a lower risk of developing type 2 diabetes^4^. Vegetarian diets are also often associated with a lower body mass index (BMI) and lower overall cancer rates^4^. Therefore, consumption of a vegetarian meal (VM) is becoming a trend and represents a good choice for people who want to maintain their health^1^. VMs are also accepted by many people for religious reasons. However, due to different religious doctrines, ingredients in the VM diet vary widely. In general, animal flesh and derivatives is excluded in the VM diet. Milk and eggs are allowed, but are not consumed in large amounts by vegetarians^5^. Thus, some religious meals or products for vegetarians are presented and sold in a market or restaurant. For example, a Buddhist vegetarian meal may be referred to as Asian strict vegetarian meal (ASVM) or an oriental vegetarian meal. In addition to the exclusion of animal ingredients including meat, milk and eggs, it is also forbidden for an ASVM to use aromatic or spicy plants or vegetables and their related flavours. These forbidden ingredients, including garlic (*Allium sativum*), Chinese leek (*Allium tuberosum*), Chinese onion (*Allium senescens*), green onion (*Allium fistulosum*), and onion (*Allium cepa*), are known as the “five forbidden vegetables” (FFVs) and are not allowed in an ASVM, even though they are considered vegetables and not animal ingredients. FFVs such as garlic, Chinese leek or green onion are commonly used in many cuisines around the world. These are generally used as a base flavouring in a cooked meal or in processed foods. As consumers including vegetarians rely on food labels to manage specific ingredients in their diet, the related information on FFVs contained in the meal should be mentioned or provided by the manufacturer to reduce consumer uncertainty and to protect consumers’ interests^6^.

A well-established nucleic acid amplification method termed loop-mediated isothermal amplification (LAMP) was first developed by Notomi *et al*. in 2000^7^. At least four primers, including a pair of outer primers and a pair of inner primers, are used to specifically recognise six distinct DNA sequences on the target gene. *Bst* DNA polymerase is used, and the LAMP reaction is performed under isothermal conditions. Due to the activity of *Bst* DNA polymerase on DNA strand displacement, DNA denaturation at a high temperature is not required during the LAMP reaction. Thus, a thermal cycler such as a PCR machine is not needed when LAMP is used. At present, LAMP is extensively applied to amplify DNA for detection or diagnostic purposes. For example, many pathogens such as viruses, fungi, bacteria, and parasites are examined using the LAMP assay^8^,^9^,^10^,^11^. Other bio-resource materials such as genetically modified organisms (GMOs), as well as animals or plants can be applied as detection targets using the LAMP assay^12^,^13^,^14^,^15^. Based on results from previous reports on the detection or identification by the LAMP method, it is considered a potential assay for the rapid, sensitive and specific detection of a bio-sample.

Polymerase chain reaction (PCR) is currently the most widely used molecular method for DNA amplification. However, very few molecular methods used to identify FFV species have been described. Only one previous official document from the Taiwan Food and Drug Administration (TFDA) reported that FFVs can successfully be detected using PCR^16^. However, some disadvantages, such as the amount of time required, assay sensitivity and integrity of the template DNA, that may reduce the effectiveness of the PCR assay were mentioned. Herein, isothermal DNA amplification was applied for the development of a LAMP assay for the rapid, sensitive and specific identification of FFVs. Additionally, the cross-reactivity of LAMP primers, integrity of the template DNA and commercially processed food products containing FFVs were examined to assess the feasibility of the established LAMP method for the identification of FFVs. To the best of our knowledge, this is the first report to identify FFVs using isothermal DNA amplification.

## Results

### Development of LAMP primers for the authentication of FFVs

**Figure 1:**
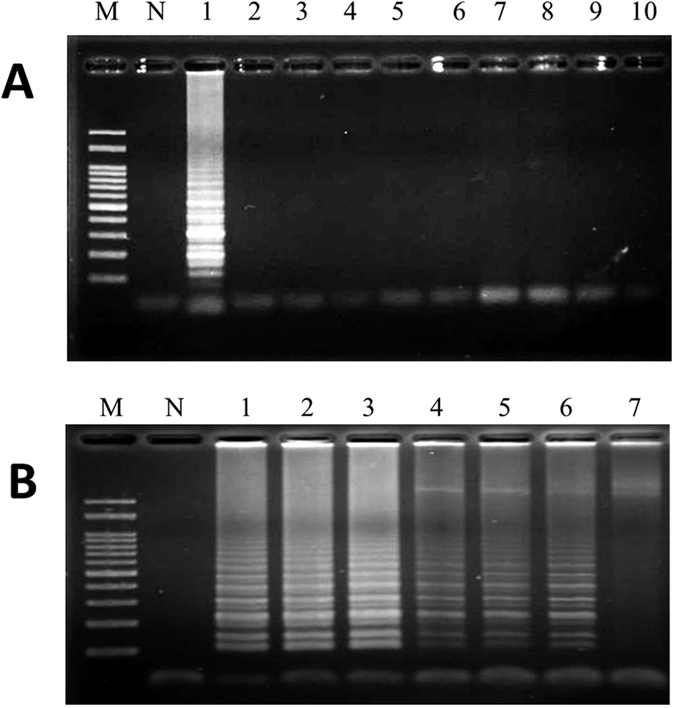
Specificity and reactivity of the LAMP assay for the identification of garlic. Purified plant genomic DNAs from garlic and other samples were used to perform LAMP. Lanes M and N represent 100 bp of DNA ladder and the negative control, respectively. (**A**) The specificity of LAMP primers used for the detection of garlic. Lanes 1–10 represent different DNAs: 1, garlic; 2, Chinese leek; 3, Chinese onion; 4, green onion; 5, onion; 6, pepper; 7, basil; 8, parsley; 9, chili; and 10, ginger. (**B**) The reactivity of LAMP primers used for the detection of garlic mixed with different percentages of the four other forbidden vegetables. Lane 1: positive control, Lanes 2–7: 50%, 20%, 10%, 5%, 2%, and 1% of garlic DNA mixed with DNAs from the four other forbidden vegetables. The adulteration ratios of FFVs are listed in Table 2.

To detect FFVs by LAMP, five sets of LAMP primers, including F3, B3, FIP and BIP, were designed to specifically identify garlic, Chinese leek, Chinese onion, green onion and onion, respectively, based on the ITS1-5.8S-ITS2 nuclear ribosomal DNA sequences available in GenBank (http://www.ncbi.nlm.nih.gov) (Table 1). When garlic LAMP primers were used for garlic identification, a typical pattern of LAMP products with ladder-like DNA fragments was revealed (Fig. 1A, lane 1). With respect to the four other LAMP primer sets for the identification of Chinese leek, Chinese onion, green onion, and onion, our results also showed that LAMP products were detected when their target DNAs were present in the samples (lane 1 of Supplementary Figs 1A to 4A). In contrast, no LAMP product was detected on an electrophoresed gel in the non-target genomic DNA samples (lanes 2 to 10 of Fig. 1A & lanes 2 to 10 of Supplementary Figs 1A to 4A). In addition, when any one set of LAMP primers such as those designed to identify garlic was used, the LAMP reaction was not influenced by the genomic DNAs of garlic, Chinese leek, Chinese onion, green onion, or onion when mixed at different percentages (Fig. 1B). Similarly, DNAs obtained from the other four FFVs were respectively mixed with the others at different adulteration ratios, as shown in Table 2. LAMP primers designed to identify Chinese leek, Chinese onion, green onion, and onion maintained their high specificities (Supplementary Figs 1B to 4B). Taken together, these results demonstrate that the LAMP primers used for the identification of FFVs established herein are specific for the authentication of garlic, Chinese leek, Chinese onion, green onion, and onion, and have been validated for their use in amplifying FFV target DNAs.

### Sensitivity of LAMP for the authentication of FFVs

**Figure 2:**
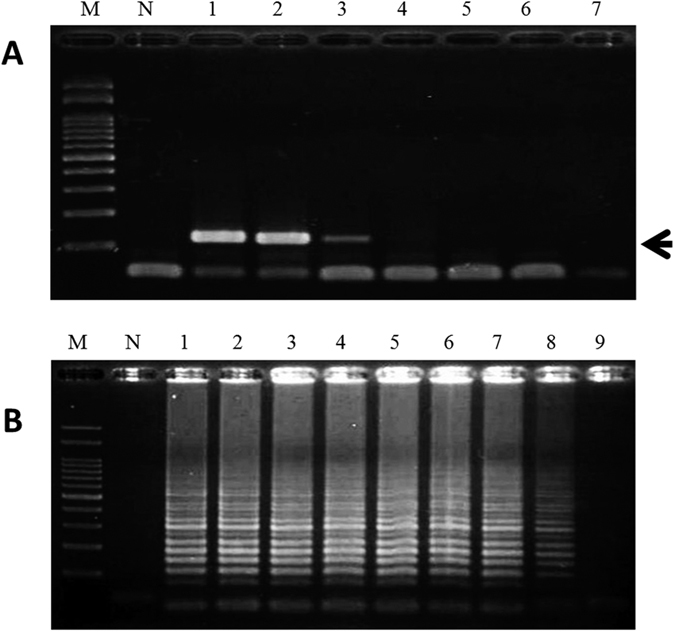
The sensitivity of specific primers used in PCR (**A**) and LAMP (**B**) for the detection of garlic. Lanes M and N represent 100 bp of DNA ladder and the negative control, respectively. Lanes 1–9 represent the addition of different amounts of garlic DNA: 1, 10^2^ ng; 2, 10^1^ ng; 3, 10^0^ ng; 4, 10^−1^ ng; 5, 10^−2^ ng; 6, 10^−3^ ng; 7, 10^−4^ ng; 8, 10^−5^ ng; and 9, 10^−6^ ng. Black arrow represents the amplified specific PCR product in the agarose gel.

To assess the sensitivity of the LAMP method, LAMP primers were used to amplify the target DNAs of garlic, Chinese leek, Chinese onion, green onion and onion. When garlic LAMP primers were used, at least 10^−5^ ng of garlic genomic DNA was required for detection (Fig. 2B). Compared to the garlic PCR primers, the amount of 1 ng (10^0^ ng) garlic genomic DNA was needed for PCR (Fig. 2A). With respect to the other four LAMP primer sets used for the authentication of Chinese leek, Chinese onion, green onion and onion, the detection limits were 10^−6^, 10^−6^, 10^−4^, and 10^−4^ ng of genomic DNA, respectively (Table 3). All four LAMP reactions were more sensitive than PCR. Taken together, these results indicate that the LAMP method established in this work for the identification of FFV DNAs is more sensitive than PCR.

### LAMP for the authentication of heat-processed FFVs

**Figure 3:**
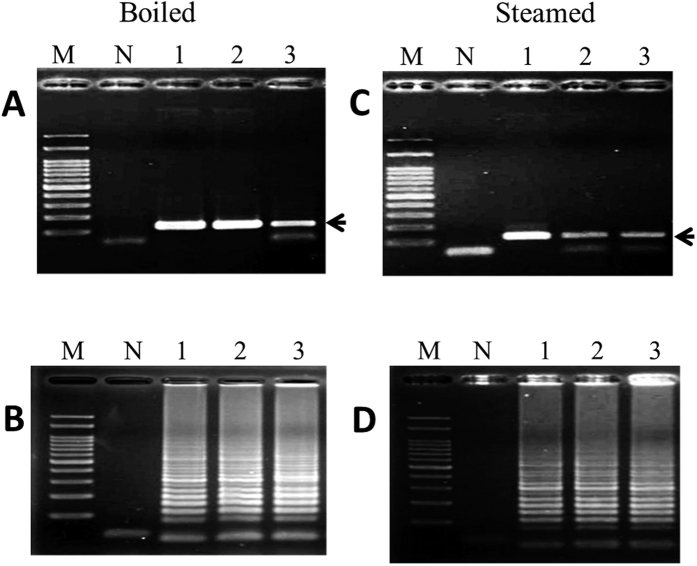
Analysis of PCR (**A,C**) and LAMP (**B,D**) products derived from garlic DNA after boiling for the indicated times. Lane M: 100 bp DNA ladder, Lane N: negative control, Lane 1: positive control (raw garlic), Lanes 2–3: garlic was boiled for 60 and 80 min and steamed for 40 and 60 min, respectively. Black arrow represents the amplified specific PCR product in the agarose gel.

**Figure 4:**
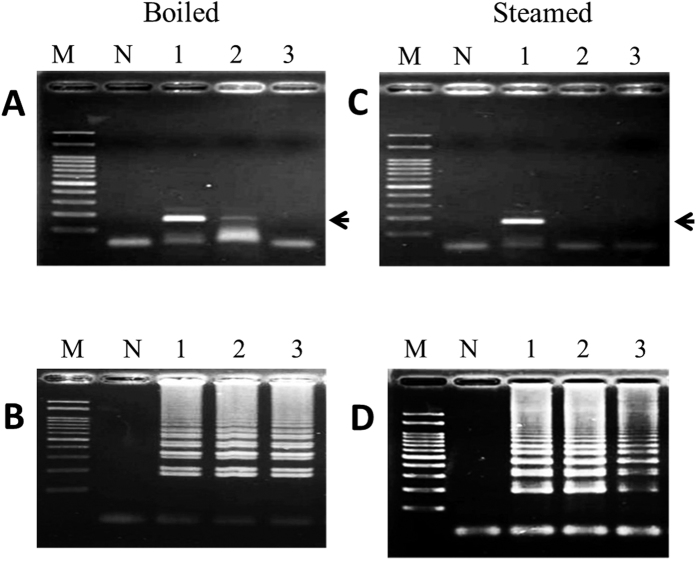
Analysis of PCR (**A,C**) and LAMP (**B,D**) products derived from onion DNA after boiling for the indicated times. Lane M: 100 bp DNA ladder, Lane N: negative control, Lane 1: positive control (raw onion), Lanes 2–3: garlic was boiled for 60 and 80 min and steamed for 40 and 60 min, respectively. Black arrow represents the amplified specific PCR product in the agarose gel.

FFVs were subjected to a boiling bath and a steam autoclave procedure. Figure 3B shows that the genomic DNA extracted from garlic following a boiling bath for 60 min or 80 min did not influence the LAMP reaction (Fig. 3B, lanes 2 & 3). With regards to the steam-autoclaved garlic DNA, products were still detected following the LAMP reaction (Fig. 3D, lanes 2 and 3). The PCR results for the detection of garlic DNA were similar to those of LAMP, regardless of whether the template DNA used in the reaction was from boiled or autoclaved garlic (Fig. 3A, lanes 2 and 3; Fig. 3C, lanes 2 and 3). Moreover, with respect to the Chinese leek, Chinese onion and green onion, extracted genomic DNAs obtained from the above mentioned heat treatments did not alter the performance of the LAMP or PCR (Supplementary Figs 5, 6 and 7). These results indicate that LAMP is well-matched in strength to PCR for the identification of garlic, Chinese leek, Chinese onion, and green onion DNAs. It is worth noting that the specific PCR products were not detected in the onion samples subjected to a boiling bath for 80 min or following any steam autoclave treatment (Fig. 4A and C). In contrast, LAMP products were detected in all heat-treated samples, including those from boiled and steam-autoclaved onions (Fig. 4B and D).

### Application of LAMP for the authentication of commercial foods containing FFVs in the market

**Figure 5:**
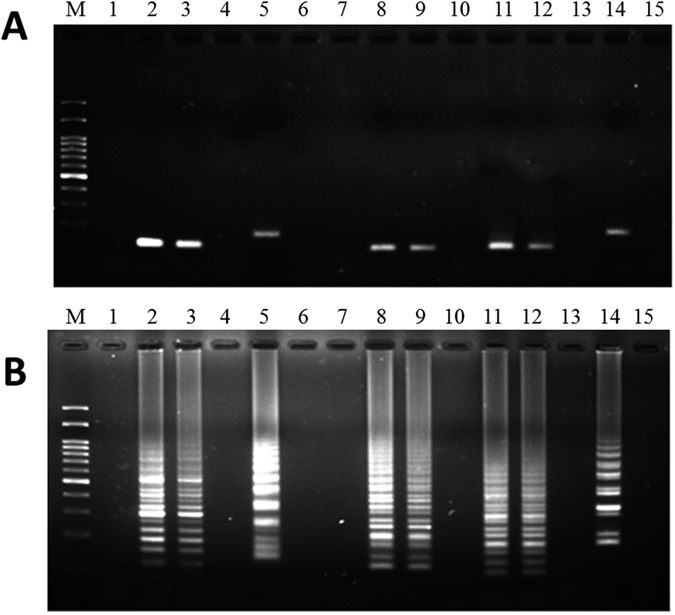
Electrophoretic analysis of PCR **(A)** and LAMP **(B)** products from commercial shacha sauce products. Lane M represents 100 bp of DNA ladder. Lanes 1, 4, 7, 10 and 13 represent the negative controls used for garlic, Chinese leek, Chinese onion, green onion, and onion primers, respectively. Lanes 2, 5, 8, 11 and 14 represent the positive controls used for the five forbidden vegetable DNAs when garlic, Chinese leek, Chinese onion, green onion, and onion primers were added to the PCR and LAMP reactions, respectively. Lanes 3, 6, 9, 12 and 15 represent the DNA products amplified by PCR and LAMP when specific primers were used for the amplification of garlic, Chinese leek, Chinese onion, green onion, and onion DNAs.

**Figure 6:**
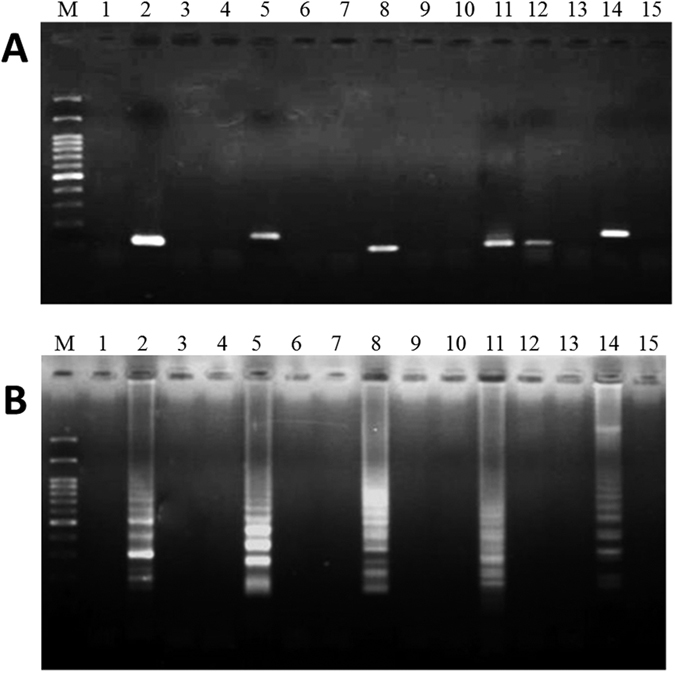
Electrophoretic analysis of PCR **(A)** and LAMP **(B)** products from commercial conditioning powder of vegetarian instant noodles products. Lane M represents 100 bp of DNA ladder. Lanes 1, 4, 7, 10 and 13 represent the negative controls used for garlic, Chinese leek, Chinese onion, green onion, and onion primers, respectively. Lanes 2, 5, 8, 11 and 14 represent the positive controls used for the five forbidden vegetable DNAs when garlic, Chinese leek, Chinese onion, green onion, and onion primers were added to the PCR and LAMP reactions, respectively. Lanes 3, 6, 9, 12 and 15 represent the DNA products amplified by PCR and LAMP when specific primers were used for the amplification of garlic, Chinese leek, Chinese onion, green onion, and onion DNAs.

To determine whether the LAMP procedure established herein is applicable as a practical tool for authenticating FFVs, three commercial foods containing FFVs and one vegetarian instant noodle were purchased from a market. The three non-vegetarian foods (shacha sauce, soda biscuit with green onion flavour and potato chips) were used to extract total DNA for use in the LAMP reaction, and five sets of respective FFV LAMP primers were used to authenticate the FFV ingredients. Figure 5B shows that at least three ingredients (garlic, Chinese onion and green onion) of FFVs were detected in the shacha sauce following LAMP. For the results of soda biscuit and potato chips, two ingredients, garlic and green onion, were detected in the soda biscuit; all three ingredients, garlic, green onion and onion, were detected in the potato chips (Supplementary Figs 8B and 9B). These results were also consistent with those from PCR detection (Supplementary Figs 8A and 9A). No FFV ingredient was detected in the conditioning powder of vegetarian instant noodles using LAMP (Fig. 6B). The majority of results obtained from LAMP were confirmed by PCR. However, it is important to note that, in the PCR on the conditioning powder of vegetarian instant noodles, green onion DNA was detected, and DNA banding was evident, albeit at a weaker intensity on the agarose gel (Fig. 6A). Taken together, these results suggest that LAMP can be used to authenticate non-vegetarian and vegetarian foods and that LAMP results agree with the labelled formulates of shacha sauce, potato chips and the conditioning powder of vegetarian instant noodles provided by the manufacturers. Only one ingredient, green onion, was detected in the soda biscuit. However, garlic DNA was amplified in all products by LAMP and PCR, indicating that the food label on the soda biscuit is mislabelled or was contaminated with garlic during food processing. In contrast, in the case of the conditioning powder of vegetarian instant noodles, the PCR results did not agree with the food label. Due to the DNA banding with a weak signal in the PCR product, the detection of green onion DNA was likely a case of incorrected food labelling or due to cross-contamination in processing. Therefore, based on the sensitivity, specificity and validation of the LAMP primers, we conclude that LAMP can be used for practical applications to authenticate FFVs to provide a more precise detection of FFVs relative to PCR.

## Discussion

A nucleic acid isothermal amplification technology has grown immensely during the past decade. Using different enzymes with DNA polymerase activity, nucleic acid amplification is performed under isothermal condition without a thermal cycler. Based on the principle of this technology, several types of isothermal amplification method has developed and used to aide in determining the bio-sample such as loop-mediated isothermal amplification (LAMP), rolling circle amplification (RCA), cross-priming amplification (CPA) or helicase dependent amplification (HDA), etc.^13^,^17^,^18^,^19^. As to LAMP, published literature has reported that LAMP provides a more convenient way to identify organisms such as bacteria, fungi, viruses, animals, and plants^8^,^9^,^11^,^12^,^13^. In this work, the specific aim was to develop a sensitive, specific and rapid method to detect FFVs. Very few studies have reported that FFVs can be identified using a molecular DNA technique such as PCR. However, only one available method is not sufficient to satisfy all conditions if met with some difficulties, such as in the purity of the DNA sample, a lower sensitivity or specificity, or a requirement for on-site operation during the identification. Thus, it is essential to develop an alternative way to more precisely and effectively overcome the weaknesses of PCR for the detection of FFVs.

In this context, LAMP primers were designed to specifically identify FFVs based on the sequences of the internal transcribed spacer (ITS) of ribosomal DNA. In our previous result, the ITS1-5.8S -ITS2 nuclear ribosomal DNA sequences of five FFVs, including garlic (*Allium sativum*), Chinese leek (*Allium tuberosum*), Chinese onion (*Allium senescens*), green onion (*Allium fistulosum*), and onion (*Allium cepa*), showed identity ranging from 75% to 95% based on sequence alignment. Thus, for successful identification of FFVs, it is important to design LAMP primers that are highly specific. Here, we successfully developed five sets of LAMP primers that can be used for the sensitive, specific and rapid identification of FFVs. With regards to the principle of primer design, each inner primer targets three distinct DNA regions for primer annealing. Therefore, the specificity of DNA amplification is higher than traditional PCR. Previous research has demonstrated that even a single nucleotide difference in DNA can be detected; therefore, the specificity of the LAMP primer is still extremely high^20^. In terms of the formation of the loop-medicated secondary DNA structure, a higher amount of DNA template is not needed for nucleic acid amplification. Therefore, the sensitivity of LAMP is also more sensitive than PCR. In this context, FFVs in the boiled and steamed foods were also detected by LAMP. Because of the lower amount of template DNA required, only a small amount of intact amplicons resulting from severe deconstruction of the DNA existed, and did not affect the reactivity of the LAMP assay. In contrast, PCR is limited by the integrity of the DNA and the copy number of amplicons, especially on template DNA originating from different foods. Due to the high sensitivity of the LAMP method, the frequency of false-positive results is perhaps higher than traditional PCR. Based on this reason, some researchers have reported that it is necessary to very carefully avoid DNA contamination while performing LAMP to decrease the number of false-positive results^21^.

To make LAMP applicable for on-site identification, it is important to determine how the data obtained from the method are presented. At least four methods can be used to examine LAMP results; these include DNA electrophoresis, visualization of SYBR green I-stained DNA by the naked eye, the observation of precipitates of magnesium pyrophosphate (MPP) and the determination of turbidity resulting from MPP^22^. In our case, when the LAMP assay for garlic identification was performed at different reaction times, LAMP products were detected and visualised conveniently by use of a fluorescent dye (Supplementary Fig. 10). Thus, once FFVs are identified by LAMP, SYBR green I staining is the first choice rather than DNA electrophoresis since the colour change is easy to observe.

Generally speaking, conventional PCR needs a longer reaction time than LAMP. In contrast, LAMP does not require a denaturation step, which differs from traditional PCR, and can thus save more time. Moreover, in terms of improving the designation of LAMP primers or the addition of a loop primer, the efficiency of LAMP can be increased significantly^10^. In Supplementary Fig. 10, LAMP primers designed for garlic DNA amplification exhibited the highest efficiency when amplifying a nucleic acid within 15 to 30 min. During this period, the intensity of fluorescence was illuminated enough to be visualised by the tester. Once the LAMP reaction was completed after 60 min, the intensity of fluorescence was almost saturated. Thus, use of a real-time detection system may be needed to precisely determine the optimal reaction time. Finally, it would be worthwhile to examine the application of the LAMP method for the authentication of FFVs to confirm ingredients in foods, especially for individuals on a vegetarian diet. The FFVs in non-vegetarian or vegetarian foods can be identified by the LAMP method established herein. Using these validated LAMP primers, even other DNAs obtained from related species of *Allium* can be mixed, while not affecting the specificity of the LAMP reaction. These results show that the food labels were in compliance with the food formulas provided by the manufacturer. Thus, use of LAMP for the identification of FFVs can be applied for internal auditing by a manufacturer to avoid contamination, or can be used for routine inspections by government authorities or for the diagnosis of a food allergen by a hospital^23^,^24^,^25^.

In summary, this study developed a molecular technique operating under isothermal conditions for the sensitive, specific and rapid identification of FFVs. This method can be applied on-site for the verification of FFVs containing vegetarian foods from the supermarket to meet the criteria of a vegetarian diet.

## Methods

### Plalnt Samples

The plant samples of garlic (*Allium sativum*), Chinese leek (*Allium tuberosum*), Chinese onion (*Allium senescens*), green onion (*Allium fistulosum*), onion (*Allium cepa*), ginger (*Zingiber officinale*), basil (*Ocimum basilicum*), parsley (*Petroselinum crispum*), chili pepper (*Capsicum annuum*) and pepper (*Piper nigrum*)were collected from local supermarket (Pingtung, Taiwan). The taxonomic identities of these plant samples were identified by Professor Chao-Lin Kuo of the China Medical University (Taichung, Taiwan), and the plant voucher specimens were deposited at the department of food science of National Pingtung University of Science and Technology.

### DNA Extraction

Total genomic DNA from plant sample of garlic, Chinese leek, Chinese onion, green onion, onion, ginger, basil, parsley, chili pepper and pepper were extracted by the genomic extraction kit (AxyPrep Multisource genomic DNA minipre kit, Axygen Bioscience, CA, USA) according to the manufacturer’s instructions. The concentration of extracted plant genomic DNA was measured by spectrophotometer (NanoVue™, GE Healthcare, Piscataway, NJ, USA). Samples were stored at −20 °C until required.

### Design of LAMP Primers

The designs of the LAMP primers (F3, B3, FIP and BIP) for authenticating garlic (*Allium sativum*), Chinese leek (*Allium tuberosum*), Chinese onion (*Allium senescens*), green onion (*Allium fistulosum*) and onion (*Allium cepa*) were based on the ITS1-5.8S-ITS2 nuclear ribosomal DNA sequence obtained from GenBank (http://www.ncbi.nlm.nih.gov) using Primer Explorer V3 software (http://primerexplorer.jp; Eiken Chemical Co., Ltd., Tokyo, Japan). The accession number of ITS1-5.8S-ITS2 nuclear ribosomal DNA sequence of garlic, Chinese leek, Chinese onion, green onion and onion are presented as AJ411901, AJ411914, AJ411834, AJ411918 and AJ411944, respectively.

### LAMP Reaction

The LAMP reaction was performed in a reaction mixture, as described previously^14^. Briefly, the reaction mixture contained 1 × *Bst* DNA polymerization buffer, 1 U of *Bst* DNA polymerase (New England Biolabs, Frankfurt, Germany), 0.5 μM outer primers (F3 and B3 primers each), 4 μM inner primers (FIP and BIP primers each), and 200 μM dNTPs each. Finally, the different amount of sample’s genomic DNA was used to each LAMP reaction. The mixtures were reacted at 60–65 °C for 60 min in a heating block (DNA engine, Biorad, CA, USA).

### Detection of LAMP Product

The detection of LAMP product was performed using DNA electrophoresis and observation of fluorescence illumination, as described in a previous study^13^. The LAMP products were directly detected by observing the color change when the diluted stock SYBR Green I reagent (Invitrogen, CA, USA), 1:10,000, was added to the end of LAMP reaction.

### PCR

The extracted genomic DNA of garlic, Chinese leek, Chinese onion, green onion and onion was used as template in the polymerase chain reaction (PCR) mixture. PCR was carried out according to previously official protocol^16^ using official designed primers (Supplementary Table 1). The PCR product was electrophoresed by 2% agarose gel and detected by observation of the presence of visible DNA bands after ethidium bromide staining.

### Preparation of boiled and steamed FFVs

Fresh plants of FFVs were obtained from a local market and cut into pieces and then weighted 2 gram for each sample to further treats by boiling and steaming, respectively. For boiling FFVs, the FFVs were boiled in hot water with 95 °C for 60 min and 80 min, respectively. Then the boiled FFVs were cooled in the ice bath and used to extract genomic DNA according to previous described procedure for performing PCR and LAMP. As to steaming of FFVs, FFVs were steamed using autoclave. The pieces of FFVs were autoclaved at 121 °C for 40 and 60 min (under pressure of 15 psi), respectively. At the end of autoclaving, the steamed FFVs were were cooled in the ice bath then subjected to genomic DNA extraction for performing PCR and LAMP.

## Additional Information

**How to cite this article**: Lee, M.-S. *et al*. The development of loop-mediated isothermal amplification (LAMP) assays for the rapid authentication of five forbidden vegetables in strict vegetarian diets. *Sci. Rep.* **7**, 44238; doi: 10.1038/srep44238 (2017).

**Publisher's note:** Springer Nature remains neutral with regard to jurisdictional claims in published maps and institutional affiliations.

## Supplementary Material

Supplementary Table and Figures

## Figures and Tables

**Table 1:** Sequences of the LAMP primers used for the detection of the five forbidden vegetables.

Species	Primers	Sequences(5′ → 3′)
Garlic	Asa_F3	TGCGACACTTGGTGTGAAT
	Asa_B3	ACTGGCAACGACAATCACTT
	Asa_FIP	CGTGCACTCGACCTAATGGCC-CCCGTGAACCATCGAGTCT
	Asa_BIP	TTCCAATCTCCCTCATGCGACG-GCACGGAAGGTCATTCTCC
Chinese leek	Atu_F3	CACACGTCATTCTAAACATCC
	Atu_B3	TGGTTTCTATTACTGTAAATCGT
	Atu_FIP	ACTTAAACCAACCGCACCTCAA-GCTTGGTAGTAATGGATATGGAG
	Atu_BIP	GATGGTTGTTGCTAGGTTTGCA-GCTAGGACTCTTGTACGC
Chinese onion	Ase_F3	AAGCGTTTTTGCTGTCAG
	Ase_B3	GATATCCATTGCCAGGAGT
	Ase_FIP	GCCGGTCTTTTATTTCTACTCTACT-TTGCGTTGTTTAGATGGGTT
	Ase_BIP	TGTGCCAAGGATAGTCGTTGTT-CATTCAGACCTATTTGCTTGT
Green onion	Afi_F3	AAATGACTCCTGGCAATGGA
	Afi_B3	TTACCGTAGGTGGGTGGTT
	Afi_FIP	CGATGGTTCACGGGATTCTGCA-CTCGTGTCGATGAAGAACGT
	Afi_BIP	ATGCAAGTTGCGCTCGAGGC-AGAATGACGCAAGGCATGA
Onion	Ace_F3	GGTTTGTGCCAAGGACAGTT
	Ace_B3	ACAGACGTGCTCTCAACCTA
	Ace_FIP	GCCAGGAGTCATTCAGACGCTC-GTTGTTGGAGAGCTTGCCAT
	Ace_BIP	AGCGAAATGCGACACTTGGTGT-GCGCAACTTGCATTCAAAGA

**Table 2:** Adulteration ratios of the five forbidden vegetables used in this study.

Target sample^a^ (%)	Garlic	Chinese leek	Chinese onion	Green onion	Onion
100	S (100%)	L (100%)	C (100%)	G (100%)	O (100%)
50	S (50%)	S (12.5%)	S (12.5%)	S (12.5%)	S (12.5%)
	L (12.5%)	L(50%)	L (12.5%)	L (12.5%)	L (12.5%)
	C (12.5%)	C (12.5%)	C (50%)	C (12.5%)	C (12.5%)
	G (12.5%)	G (12.5%)	G (12.5%)	G (50%)	G (12.5%)
	O (12.5%)	O (12.5%)	O (12.5%)	O (12.5%)	O (50%)
20	S (20%)	L (20%)	C (20%)	G (20%)	O (20%)
10	S (10%)	S (22.5%)	S (22.5%)	S (22.5%)	S (22.5%)
	L (22.5%)	L (10%)	L (22.5%)	L (22.5%)	L (22.5%)
	C (22.5%)	C (22.5%)	C (10%)	C (22.5%)	C (22.5%)
	G (22.5%)	G (22.5%)	G (22.5%)	G (10%)	G (22.5%)
	O (22.5%)	O (22.5%)	O (22.5%)	O (22.5%)	O (10%)
5	S (5%)	S (23.75%)	S (23.75%)	S (23.75%)	S (23.75%)
	L (23.75%)	L (5%)	L (23.75%)	L (23.75%)	L (23.75%)
	C (23.75%)	C (23.75%)	C (5%)	C (23.75%)	C (23.75%)
	G (23.75%)	G (23.75%)	G (23.75%)	G (5%)	G (23.75%)
	O (23.75%)	O (23.75%)	O (23.75%)	O (23.75%)	O (5%)
2	S (2%)	S (24.5%)	S (24.5%)	S (24.5%)	S (24.5%)
	L (24.5%)	L (2%)	L (24.5%)	L (24.5%)	L (24.5%)
	C (24.5%)	C (24.5%)	C (2%)	C (24.5%)	C (24.5%)
	G (24.5%)	G (24.5%)	G (24.5%)	G (2%)	G (24.5%)
	O (24.5%)	O (24.5%)	O (24.5%)	O (24.5%)	O (2%)
1	S (1%)	S (24.75%)	S (24.75%)	S (24.75%)	S (24.75%)
	L (24.75%)	L (1%)	L (24.75%)	L (24.75%)	L (24.75%)
	C (24.75%)	C (24.75%)	C (1%)	C (24.75%)	C (24.75%)
	G (24.75%)	G (24.75%)	G (24.75%)	G (1%)	G (24.75%)
	O (24.75%)	O (24.75%)	O (24.75%)	O (24.75%)	O (1%)

S, Garlic; L, Chinese leek; C, Chinese onion; G, Green onion; O, Onion.

^a^Adulteration ratio of FFVs were prepared by wet weight.

**Table 3:** Comparison of detection limits of the developed LAMP and PCR methods for the five forbidden vegetables.

Detection limit (ng of DNA)		
Sample	PCR	LAMP
Garlic	10^0^	10^−5^
Chinese leek	10^−2^	10^−6^
Chinese onion	10^−2^	10^−6^
Green onion	10^0^	10^−4^
Onion	10^0^	10^−4^
