# Supplementary material for: The development of loop-mediated isothermal amplification (LAMP) assays for the rapid authentication of five forbidden vegetables in strict vegetarian diets
Source: Sci Rep. 2017 Mar 14;7:44238. doi: 10.1038/srep44238 (PMC5349574; doi:10.1038/srep44238)
Supplement: Supplementary Table and Figures [file srep44238-s1.pdf]

The development of loop-mediated isothermal amplification (LAMP) assays for the rapid authentication of five forbidden vegetables in strict vegetarian diets

Meng-Shiou Lee[1*], Ting-Ying Su[2], Yi-Yang Lien[3], Shyang-Chwen Sheu[2*]

[1] China Medical University, Department of Chinese Pharmaceutical Science and Chinese Medicine Resources, Taichung, 40402, Taiwan

[2] National Pingtung University of Science and Technology, Department of Food Science, Pingtung, 91201, Taiwan

[3] National Pingtung University of Science and Technology, Department of Veterinary Medicine, Pingtung, 91201, Taiwan

*****Authors to whom correspondence should be addressed to M.-S.L. (leemengshiou@mail.cmu.edu.tw) or S.-C.S. (ssheu@mail.npust.edu.tw)

Supplementary tables

Supplementary table S1. Sequences of PCR primers and size of PCR product for the detection of five forbideen vegetables[16].

Species	Primers	Sequences (5’---3’)	Size of PCR product
Garlic	AsF<br>AsR	CGACGAGTGCATTTTGGGTTATGATG<br>CATCGTGCGTTAACTCGACACACCAT	130 bp
Chinese leek	AtF<br>AtR	CACGTCATTCTAAACATCCATC<br>TTGTACGCAGTTAGAGATCGCG	170 bp
Chinese onion	AcF<br>AcR	CGACAAACGTATCGTGGG<br>TCGATACACCATTCGCCG	117 bp
Green onion	GROF<br>GROR	CGGTGCAAGGTGGAACATTCGTCCA<br>TTACCATCCCTGGACGGTGGTG	117 bp
Onion	ONIF<br>ONIR	TCTAGATGTCGCATCAGTGGAATCC<br>CGTGACAACTCCTCTTCC	174 bp

Supplementary figures

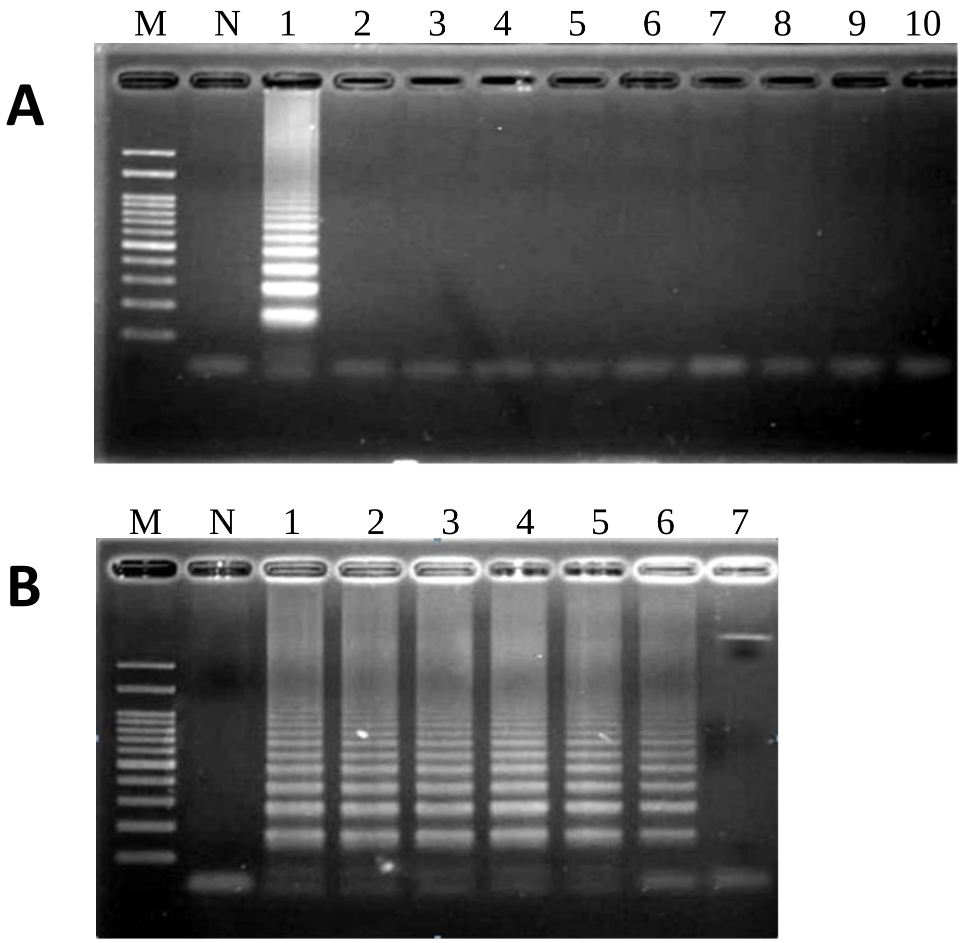

Supplementary Figure S1. Specificity and reactivity of the LAMP assay for the identification of Chinese leek. Purified plant genomic DNAs from garlic and other samples were used to perform LAMP. Lanes M and N represent 100 bp of DNA ladder and the negative control, respectively. (**A**) The specificity of LAMP primers used for the detection of garlic. Lanes 1-10 represent different DNAs: 1, Chinese leek; 2, garlic; 3, Chinese onion; 4, green onion; 5, onion; 6, pepper; 7, basil; 8, parsley; 9, chili; and 10, ginger. (**B**) The reactivity of LAMP primers used for the detection of garlic mixed with different percentages of the four other forbidden vegetables. Lane 1: positive control, Lanes 2-7: 50%, 20%, 10%, 5%, 2%, and 1% of garlic DNA mixed with DNAs from the four other forbidden vegetables. The adulteration ratios of FFVs are listed in Table 2.

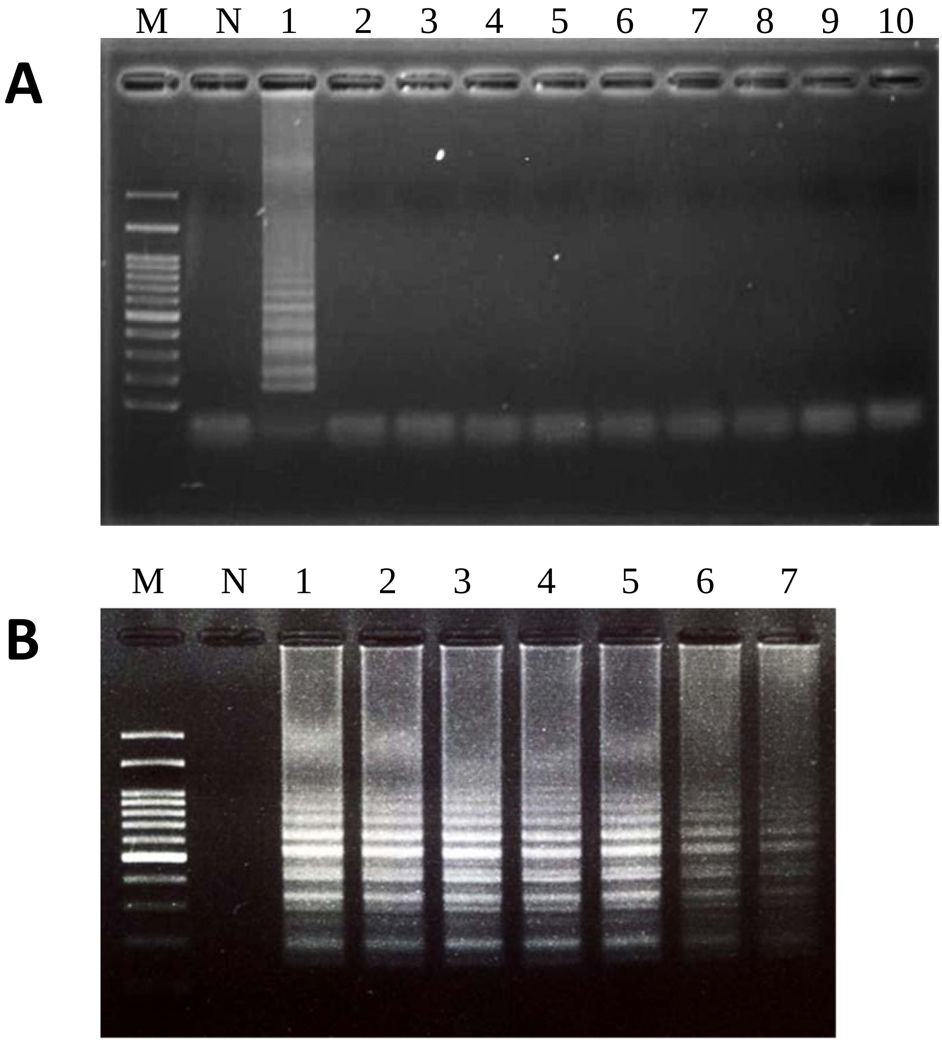

Supplementary Figure S2. Specificity and reactivity of the LAMP assay for the identification of Chinese onion. Purified plant genomic DNAs from garlic and other samples were used to perform LAMP. Lanes M and N represent 100 bp of DNA ladder and the negative control, respectively. (**A**) The specificity of LAMP primers used for the detection of garlic. Lanes 1-10 represent different DNAs: 1, Chinese onion; 2, garlic; 3, Chinese leek; 4, green onion; 5, onion; 6, pepper; 7, basil; 8, parsley; 9, chili; and 10, ginger. (**B**) The reactivity of LAMP primers used for the detection of garlic mixed with different percentages of the four other forbidden vegetables. Lane 1: positive control, Lanes 2-7: 50%, 20%, 10%, 5%, 2%, and 1% of garlic DNA mixed with DNAs from the four other forbidden vegetables. The adulteration ratios of FFVs are listed in Table 2.

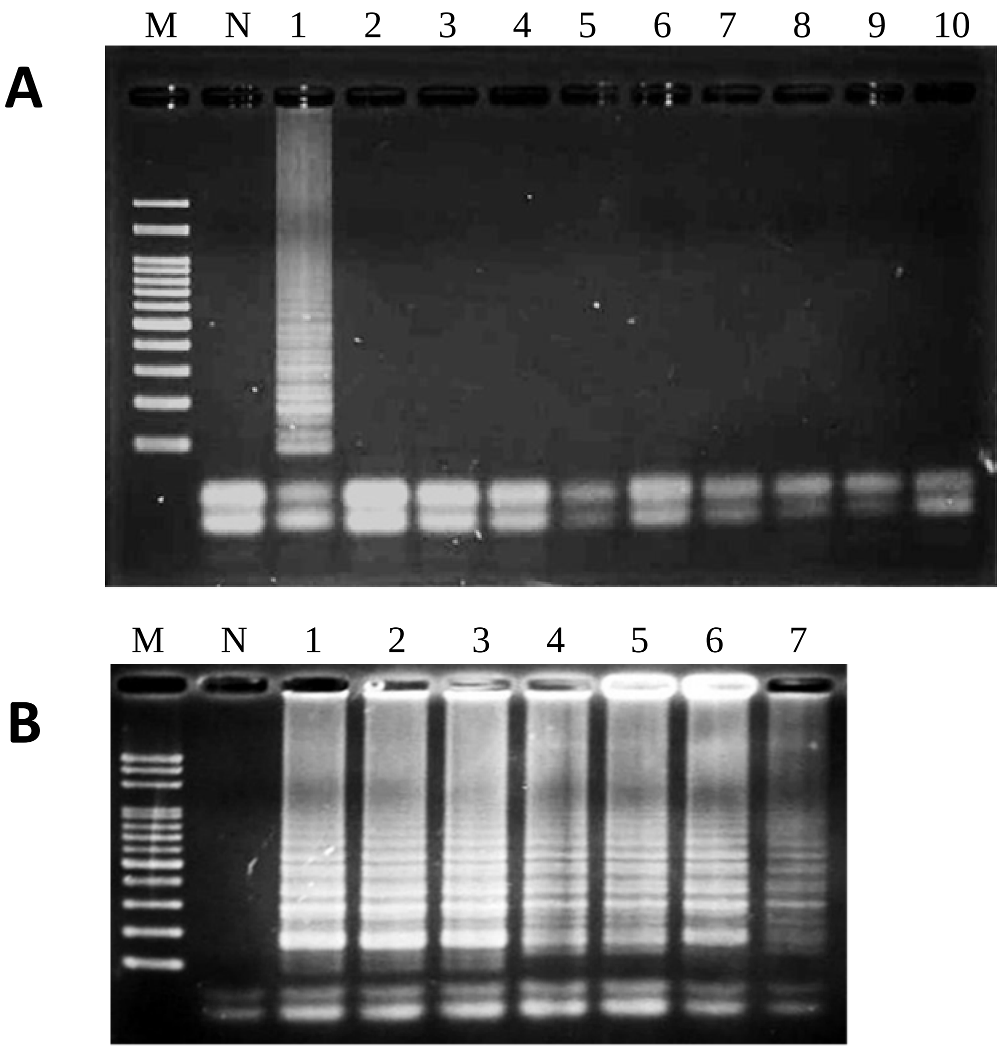

Supplementary Figure S3. Specificity and reactivity of the LAMP assay for the identification of green onion. Purified plant genomic DNAs from garlic and other samples were used to perform LAMP. Lanes M and N represent 100 bp of DNA ladder and the negative control, respectively. (**A**) The specificity of LAMP primers used for the detection of garlic. Lanes 1-10 represent different DNAs: 1, green onion; 2, garlic; 3, Chinese leek; 4, Chinese onion; 5, onion; 6, pepper; 7, basil; 8, parsley; 9, chili; and 10, ginger. (**B**) The reactivity of LAMP primers used for the detection of garlic mixed with different percentages of the four other forbidden vegetables. Lane 1: positive control, Lanes 2-7: 50%, 20%, 10%, 5%, 2%, and 1% of garlic DNA mixed with DNAs from the four other forbidden vegetables. The adulteration ratios of FFVs are listed in Table 2.

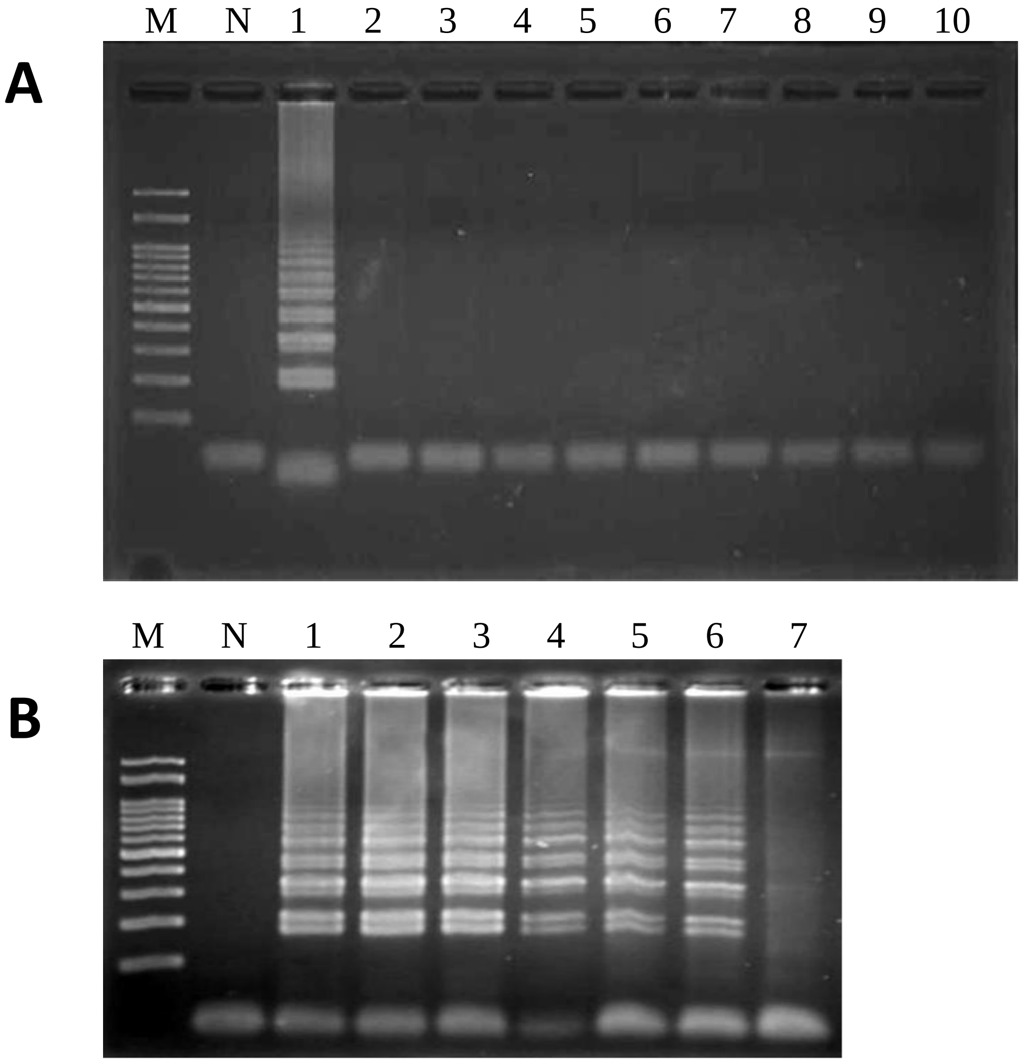

Supplementary Figure S4. Specificity and reactivity of the LAMP assay for the identification of onion. Purified plant genomic DNAs from garlic and other samples were used to perform LAMP. Lanes M and N represent 100 bp of DNA ladder and the negative control, respectively. (**A**) The specificity of LAMP primers used for the detection of garlic. Lanes 1-10 represent different DNAs: 1, onion; 2, garlic; 3, Chinese leek; 4, Chinese onion; 5, green onion; 6, pepper; 7, basil; 8, parsley; 9, chili; and 10, ginger. (**B**) The reactivity of LAMP primers used for the detection of garlic mixed with different percentages of the four other forbidden vegetables. Lane 1: positive control, Lanes 2-7: 50%, 20%, 10%, 5%, 2%, and 1% of garlic DNA mixed with DNAs from the four other forbidden vegetables. The adulteration ratios of FFVs are listed in Table 2.

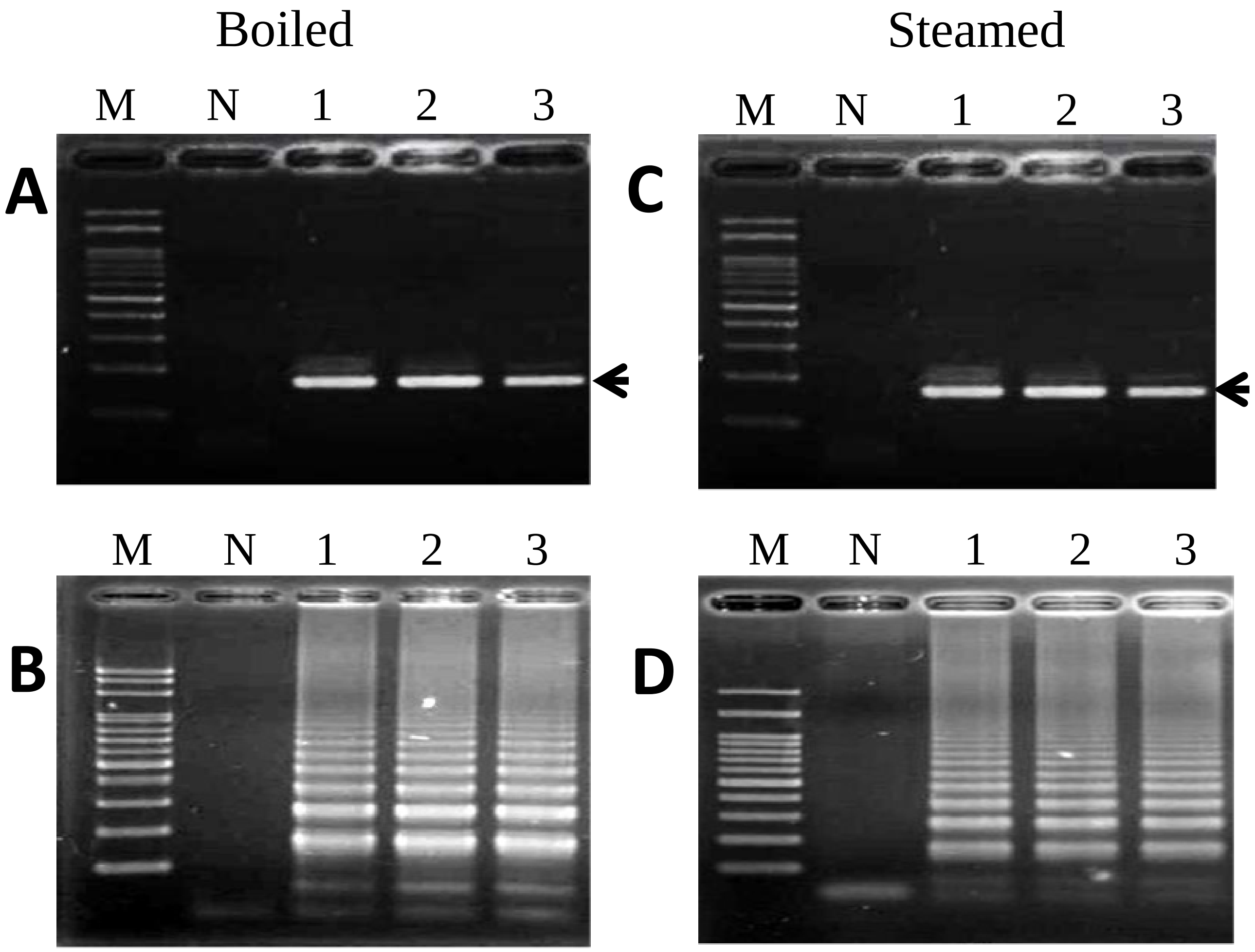

Supplementary Figure S5. Analysis of PCR (A, C) and LAMP (B, D) products derived from Chinese leek DNA after boiling for the indicated times. Lane M: 100 bp DNA ladder, Lane N: negative control, Lane 1: positive control (raw Chinese leek), Lanes 2-3: Chinese leek was boiled for 60 and 80 min and steamed for 40 and 60min, respectively. Black arrow represents the amplified specific PCR product in the agarose gel.

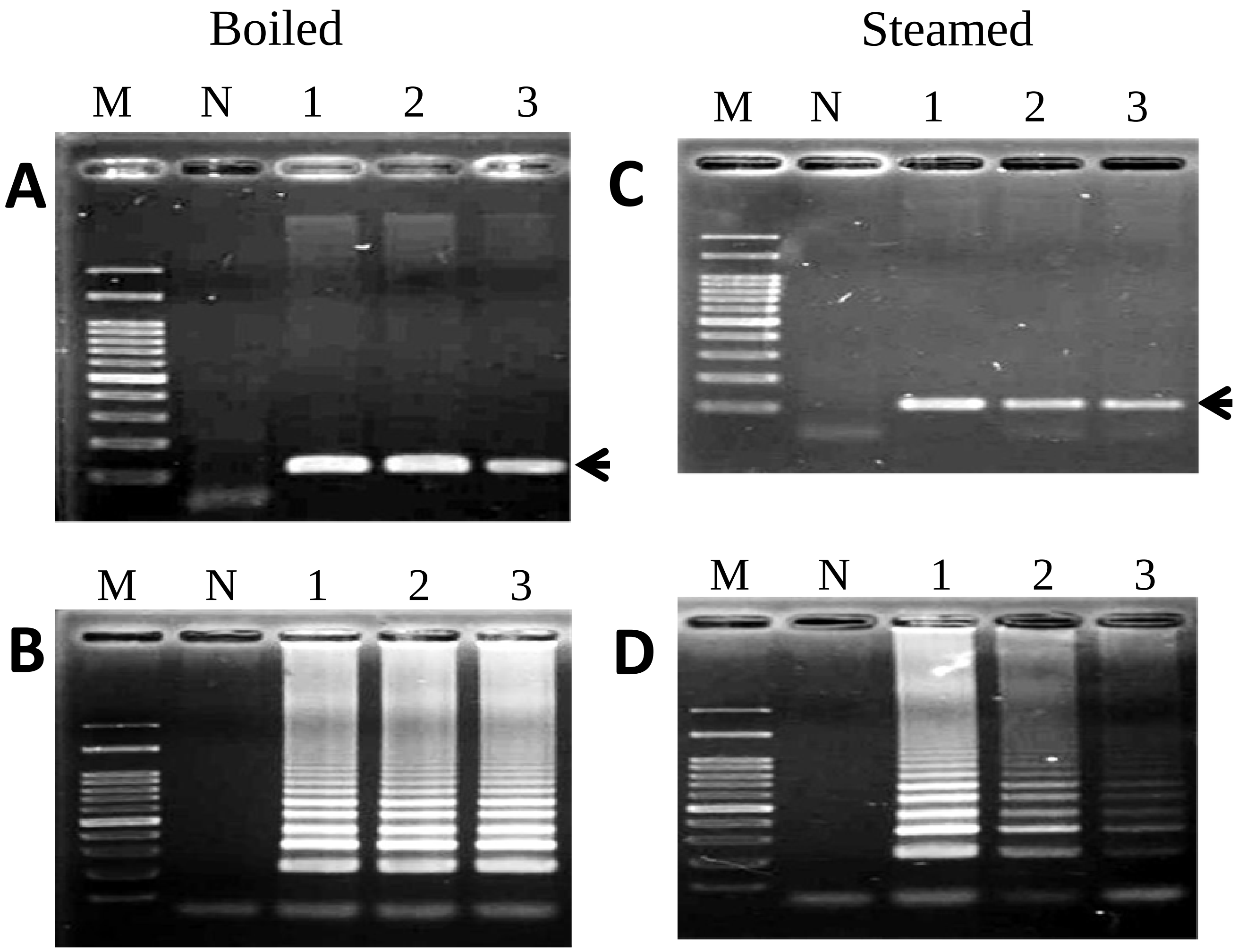

Supplementary Figure S6. Analysis of PCR (A, C) and LAMP (B, D) products derived from Chinese onion DNA after boiling for the indicated times. Lane M: 100 bp DNA ladder, Lane N: negative control, Lane 1: positive control (raw Chinese onion), Lanes 2-3: Chinese onion was boiled for 60 and 80 min and steamed for 40 and 60min, respectively. Black arrow represents the amplified specific PCR product in the agarose gel.

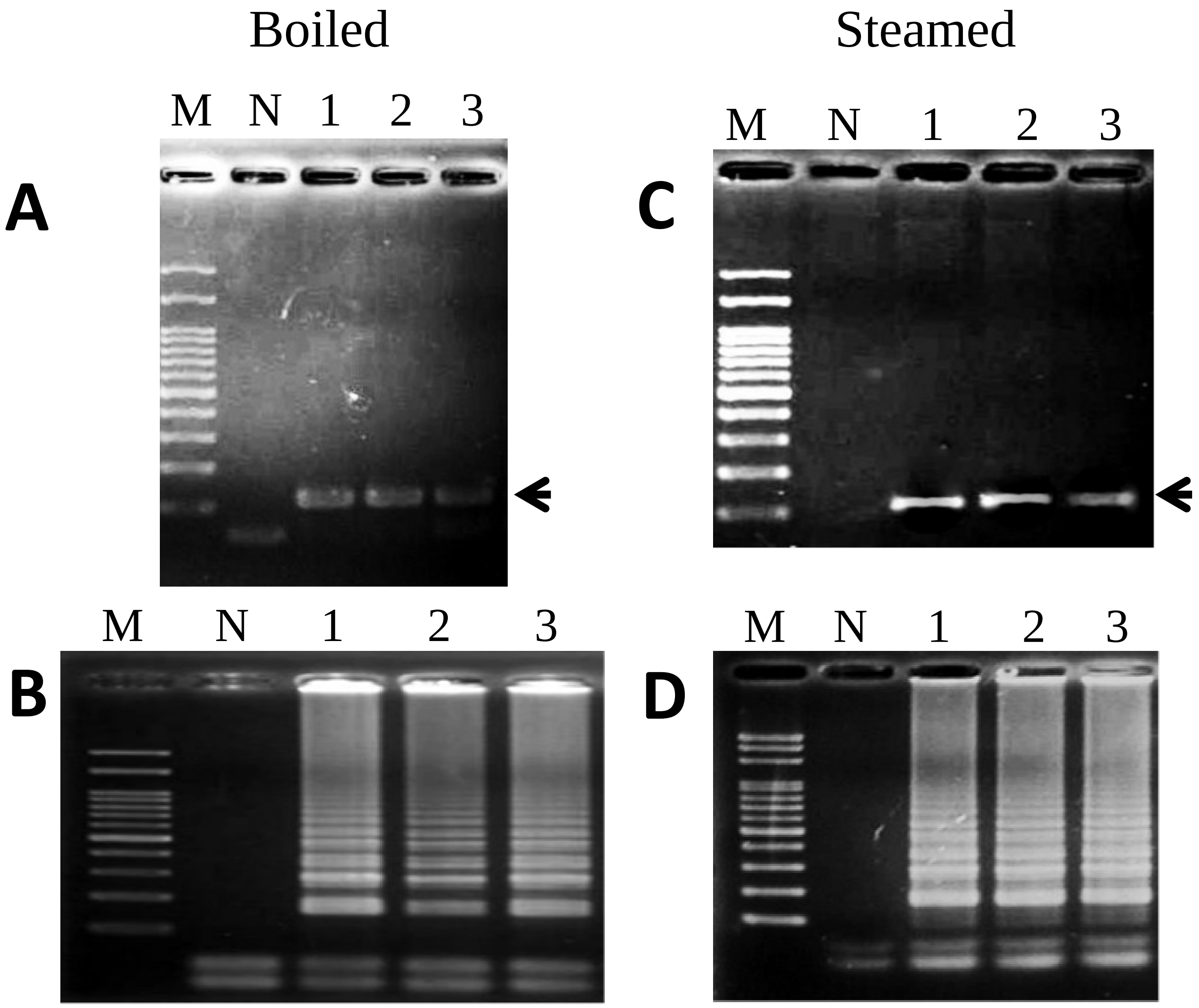

Supplementary figure S7. Analysis of PCR (A, C) and LAMP (B, D) products derived from green onion DNA after boiling for the indicated times. Lane M: 100 bp DNA ladder, Lane N: negative control, Lane 1: positive control (raw green onion), Lanes 2-3: green onion was boiled for 60 and 80 min and steamed for 40 and 60min, respectively. Black arrow represents the amplified specific PCR product in the agarose gel.

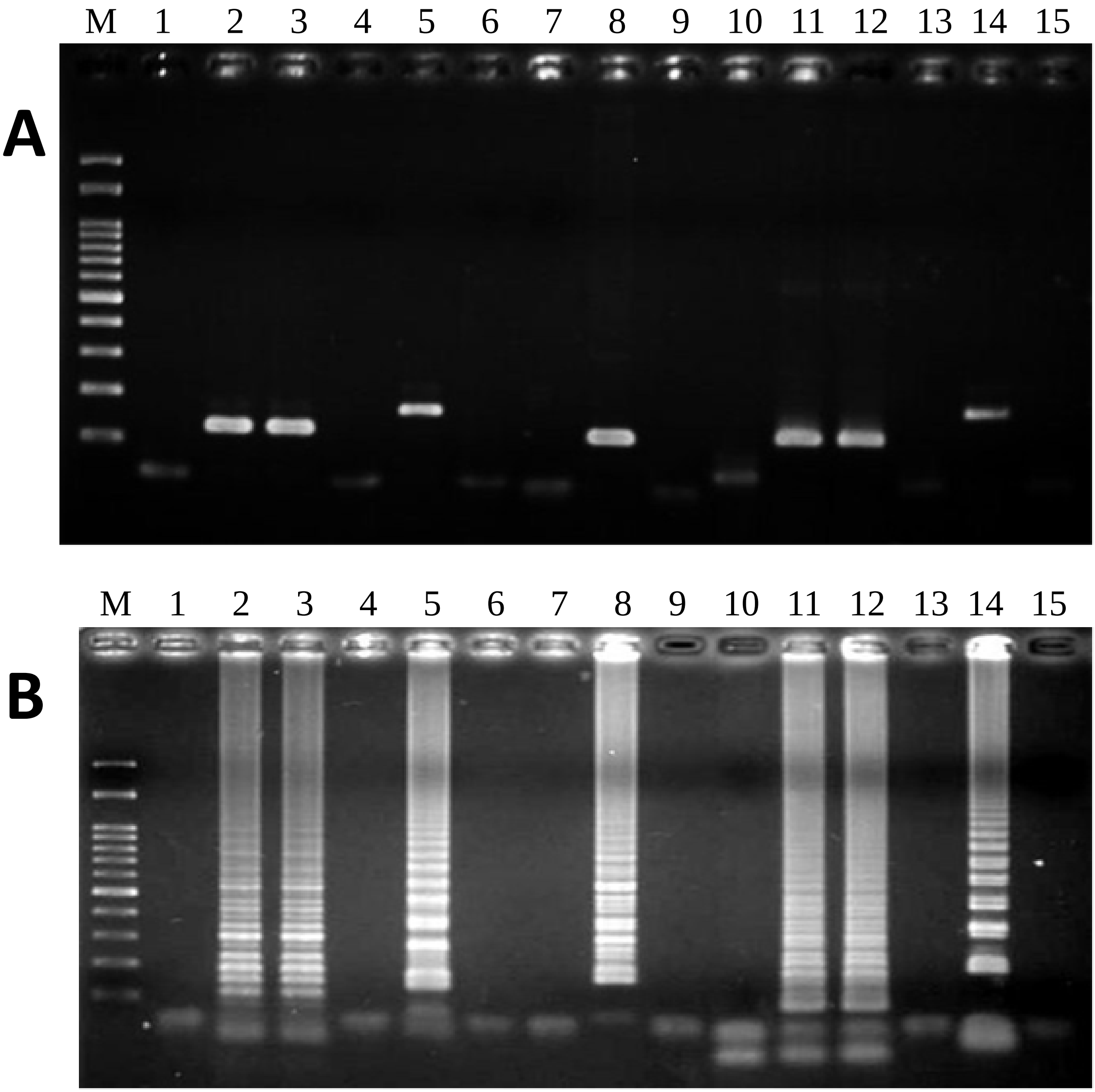

Supplementary figure S8. Electrophoretic analysis of PCR **(A)** and LAMP **(B)** products from commercial soda biscuit with green onion flavour products. Lane M represents 100 bp of DNA ladder. Lanes 1, 4, 7, 10 and 13 represent the negative controls used for garlic, Chinese leek, Chinese onion, green onion, and onion primers, respectively. Lanes 2, 5, 8, 11 and 14 represent the positive controls used for the five forbidden vegetable DNAs when garlic, Chinese leek, Chinese onion, green onion, and onion primers were added to the PCR and LAMP reactions, respectively. Lanes 3, 6, 9, 12 and 15 represent the DNA products amplified by PCR and LAMP when specific primers were used for the amplification of garlic, Chinese leek, Chinese onion, green onion, and onion DNAs.

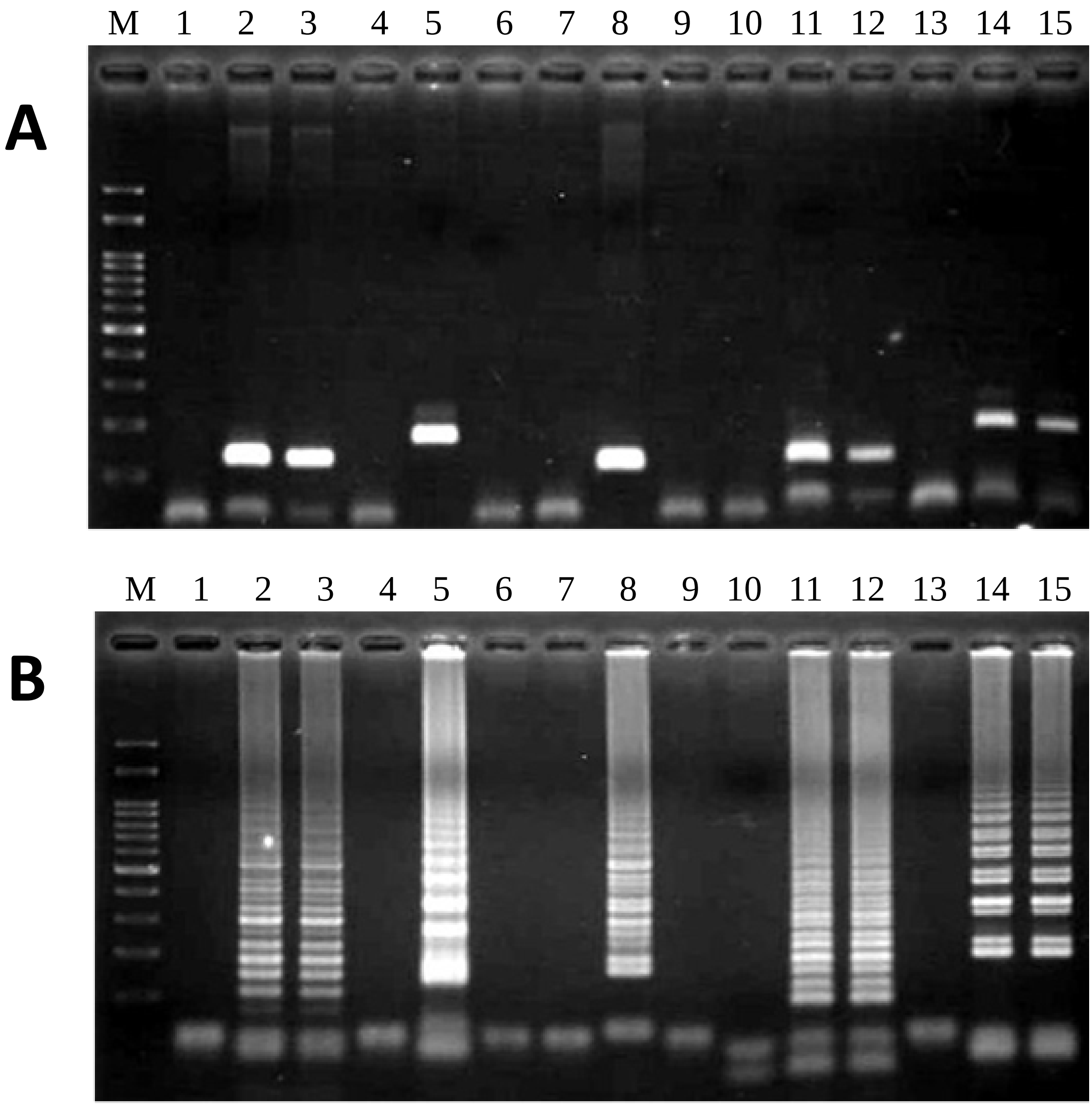

Supplementary Figure S9. Electrophoretic analysis of PCR **(A)** and LAMP **(B)** products from commercial potato chip products. Lane M represents 100 bp of DNA ladder. Lanes 1, 4, 7, 10 and 13 represent the negative controls used for garlic, Chinese leek, Chinese onion, green onion, and onion primers, respectively. Lanes 2, 5, 8, 11 and 14 represent the positive controls used for the five forbidden vegetable DNAs when garlic, Chinese leek, Chinese onion, green onion, and onion primers were added to the PCR and LAMP reactions, respectively. Lanes 3, 6, 9, 12 and 15 represent the DNA products amplified by PCR and LAMP when specific primers were used for the amplification of garlic, Chinese leek, Chinese onion, green onion, and onion DNAs.

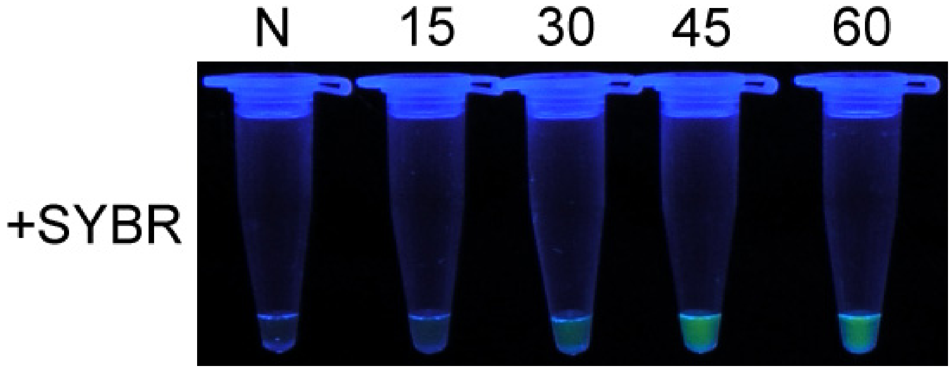

Supplementary Figure 10. Analysis of LAMP products using a fluorescent dye (SYBR Green I). Extracted garlic genomic DNA was used as the template and LAMP primers designed for garlic identification were added to the LAMP reaction. After LAMP, products were stained with SYBR Green I and visualised under UV excitation. Lane N, negative control; Lanes 15, 30, 45 and 60 represent LAMP products after 15, 30, 45 and 60 min reaction times, respectively.
